# Supplementary material for: Preventing Multimer Formation in Commonly Used Synthetic Biology Plasmids
Source: ACS Synth Biol. 2025 Mar 18;14(4):1309–15. doi: 10.1021/acssynbio.4c00508 (PMC12012879; doi:10.1021/acssynbio.4c00508)
Supplement: Supplementary file 1 — sb4c00508_si_001.pdf [file sb4c00508_si_001.pdf]

Supplemental Information for:

Preventing multimer formation in commonly used synthetic biology plasmids

Elizabeth Vaisbourd[1], Anat Bren[1], Uri Alon[1], David S. Glass[1*]

[1] Department of Molecular Cell Biology, Weizmann Institute of Science, Rehovot, Israel 76100

[*] david.glass@weizmann.ac.il

Document contains:

Figure S1

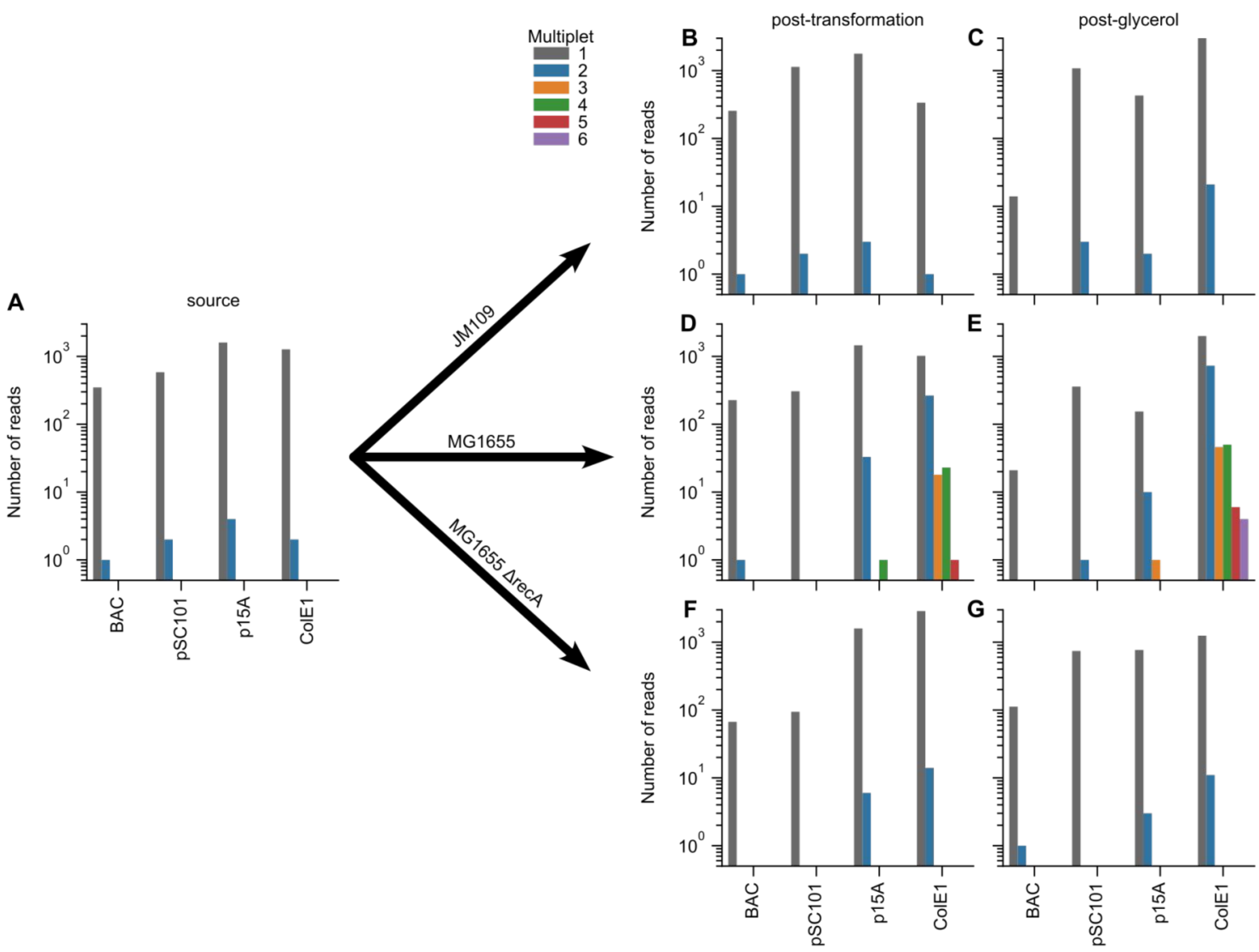

Figure S1. Mapped read counts for data from Fig. 2B-H.

A - G. Number of raw reads corresponding to data from Fig. 2B-H, respectively. Here we include the singlet counts as well.
